# Adequate vitamin B_12_ and folate status of Norwegian vegans and vegetarians

**DOI:** 10.1017/S0007114522002987

**Published:** 2023-06-28

**Authors:** Sigrun Henjum, Synne Groufh-Jacobsen, Allen Lindsay, Ellen Raael, Anna Maria Israelsson, Setareh Shahab-Ferdows, Daniela Hampel

**Affiliations:** 1 Department of Nursing and Health Promotion, Faculty of Health Sciences, Oslo Metropolitan University, Kunnskapsveien 55, 2007 Kjeller, 0130 Oslo, Norway; 2 Department of Nutrition and Public Health, Faculty of Health and Sport Science, University of Agder, Universitetsveien 25, 4630 Kristiansand, Norway; 3 USDA/ARS Western Human Nutrition Research Center, 430 West Health Sciences Drive, Davis, CA 95616, USA; 4 Department of Nutrition, University of California, One Shields Ave, Davis, CA 95616, USA

**Keywords:** Vitamin B_12_, Folate, Homocysteine, Methylmalonic acid, Vegans, Vegetarians, B_12_ dietary intake

## Abstract

Plant-based diets may increase the risk of vitamin B_12_ deficiency due to limited intake of animal-source foods, while dietary folate increases when adhering to plant-based diets. In this cross-sectional study, we evaluated the B_12_ and folate status of Norwegian vegans and vegetarians using dietary B_12_ intake, B_12_ and folic acid supplement use, and biomarkers (serum B_12_ (B_12_), plasma total homocysteine (tHcy), plasma methylmalonic acid (MMA) and serum folate). Vegans (*n* 115) and vegetarians (*n* 90) completed a 24-h dietary recall and a FFQ and provided a non-fasting blood sample. cB_12_, a combined indicator for evaluation of B_12_ status, was calculated. B_12_ status was adequate in both vegans and vegetarians according to the cB_12_ indicator; however 4 % had elevated B_12_. Serum B_12_, tHcy, MMA concentrations and the cB_12_ indicator (overall median: 357 pmol/l, 9·0 µmol/l, 0·18 µmol/l, 1·30 (cB_12_)) did not differ between vegans and vegetarians, unlike for folate (vegans: 25·8 nmol/l, vegetarians: 21·6 nmol/l, *P* = 0·027). Serum B_12_ concentration < 221 pmol/l was found in 14 % of all participants. Vegetarians revealed the highest proportion of participants below the recommended daily intake of 2 µg/d including supplements (40 *v*. 18 %, *P* < 0·001). Predictors of higher serum B_12_ concentrations were average daily supplement use and older age. Folate deficiency (< 10 nmol/l) was uncommon overall (< 2·5 %). The combined indicator cB_12_ suggested that none of the participants was B_12_-depleted; however, low serum B_12_ concentration was found in 14 % of the participants. Folate concentrations were adequate, indicating adequate folate intake in Norwegian vegans and vegetarians.

Plant-based diets can provide several health beneﬁts such as lower serum cholesterol, blood pressure and weight. However, these diets may also increase the risks of micronutrient deﬁciencies^(1–3)^.Vitamin B_12_ (B_12_) is naturally present only in animal-source foods, and people who follow a plant-based diet and do not consume sufficient quantities of foods fortified with B_12_ or take supplements will be at risk of B_12_ deficiency^(4)^. Fruits, vegetables, berries and grains are foods rich in folate^(5)^. B_12_ and folate are linked via the methyl group transfer from N^5^-methyltetrahydrofolate to B_12_, and deficiencies can lead to megaloblastic anaemia and hyperhomocysteinemia, a risk factor for atherosclerosis^(6)^. Since B_12_ is essential for DNA and RNA synthesis, erythropoiesis and the production of neurotransmitters^(7)^, low B_12_ status can cause neurological damage due to an inhibition of the formation of the myelin sheath among other potential causes^(4)^. Folate is essential for physiological processes, such as synthesis of nucleic acids and low folate status may cause cognitive and neurological symptoms. B_12_ status can be assessed using several biomarkers, such as B_12_ concentrations, total homocysteine (tHcy) and methylmalonic acid (MMA). tHcy and MMA are both functional biomarkers that accumulate when B_12_ status is poor. However, tHcy also increases with low folate status^(8)^. Folate status can be assessed by evaluating serum folate and tHcy.

Causes of B_12_ deficiency can be divided into four main types: inadequate intake from food, malabsorption, chemical inactivation by nitrous oxide or genetic disorders^(9)^. In high-income populations, B_12_ deficiency is more often diagnosed as a cause of low absorption due to pernicious anaemia, an autoimmune condition where intrinsic factor production is inadequate^(9)^. Loss of intrinsic factor also occurs with ageing. Deficiency is also more common in people who consume limited amounts of animal-source foods, such as vegans, vegetarians and the elderly^(4)^. In low- and middle-income countries, B_12_ deficiency can be relatively common due to lack of income to purchase animal-source foods or because of religious or cultural dietary preferences^(9)^. Previous research in populations consuming a mixed diet reported that serum B_12_ concentrations are on average lower in older individuals^(10)^, but few studies have included older vegans and vegetarians.

According to national dietary surveys, healthy adults in Sweden and Norway have inadequate folate intakes. Three out of four women and one out of two men have been found to have intakes below the recommended intake in Norway^(11–15)^. Vegans and vegetarians have higher intakes of fruits and vegetables than the general population, likely resulting in a higher folate status in these groups. Worldwide, fortification of flour with folic acid is common, in USA folic acid fortification of all cereal grain product flour was implemented in 1998; however in Norway, flour with folic acid fortification is not available.

Interest in plant-based diets has increased over the past few years^(16)^, and vegans now represent at least 1 % and vegetarians about 3 % of the Norwegian population^(17)^. In addition, a flexitarian diet, reducing the intake of meat, milk, eggs and fish in favour of plant-based alternatives, is becoming increasingly popular^(18)^. Given the very limited information available, this study evaluated B_12_ and folate status of vegan and vegetarian adults in Norway, using dietary B_12_ intake and supplement use of vitamin B_12_ and folic acid, and serum B_12_ concentrations to detect subclinical or clinical deﬁciency in conjunction with the functional markers tHcy, MMA and serum folate. The measured biomarkers were further used to calculate the recently proposed B_12_ status indicator, cB_12_^(19)^.

## Methods

### Participants

In this study, 205 participants, 115 vegans and 90 vegetarians, from the Oslo area, were included (57 men and 148 women, age range18–60 years). The inclusion criteria were as follows: (1) no consumption of poultry, meat and/or meat products the previous 6 months or more; (2) older than 18 years of age; (3) not pregnant or lactating; (4) no chronic or acute illness know to affect B_12_ status or acute illness.

Vegans were defined as people who omitted all types of animal-source foods from their diet, and vegetarians as those who excluded poultry, meat, and meat products, but included milk and dairy products and/or eggs and/or fish in varying degrees. Due to few pescatarians (*n* 35), and no difference in B_12_ status between vegetarians and pescatarians, the two groups were merged.

Participants were mainly recruited through social media, using convenience sampling method. The snowball effect was further used to recruit participants through existing participants. Information sheets about the study purpose and participation were shared on OsloMet’s website, a website for health personnel interested in plant-based diets (HEPLA) and in closed Facebook groups for vegans and vegetarians.

### Dietary questionnaire – habitual intake

Participants answered an electronic questionnaire, which consisted of two parts. The first part covered background information (age, height and weight, marital status, occupational status, educational level, smoking habits, country of birth, language, dietary practice, and duration of adherence to vegan/vegetarian diet), while the second part included a FFQ, assessing habitual food and supplement intake using thirty-two questions about average intake of selected foods/food groups and supplements over the past 4 weeks. These questions had seven frequency alternatives ranging from ‘rarely/never’, ‘less frequently than weekly’, ‘1–3 times per week’, ‘4–6 times per week’, ‘1–2 times per day’, ‘3–4 times a day’ to ‘5 or more times a day’. The answers were converted into daily amounts and adjusted for portion size to obtain B_12_ intake. Type, amount, brand and frequency of supplements used were also assessed for both B_12_ and folic acid. The use of B_12_ injections was assessed based on whether they ever had taken B_12_ injections and time since last injection (months).

The questionnaire was designed based on a previously validated questionnaire in a study of lactating women and iodine status^(20)^. Changes were made to adapt to vegans and vegetarian diets and relevant lifestyle factors, including several plant-based alternatives such as legumes, plant-based milk and other non-dairy products (oats, rice, soya, almond and coconut), or vegan cheese and meat substitutes (soya products, tofu and tempeh). Energy drink intake was also evaluated, due to high B_12_ doses in some of the brands. Dietary folate intake was not calculated due to insufficient details on folate sources (e.g. vegetable was one food group and did not specify which type of vegetable).

### Twenty-four-hour dietary recall

The 24-h recall was completed for B_12_ intakes on the same day as the non-fasting blood sample was collected. Types and quantities (grams and decilitres) of food and drinks, and brand or manufacturer were assessed. To calculate the 24-h intake, reported food items were multiplied by B_12_ concentrations in each specific food item available in the Norwegian Food Composition Table in 2019^(5)^. For combined food items, the mean values for each food item were used (e.g. all lean fish types, all fatty fish species, all types of cake/chocolate/ice cream and all types of vegetables). Plant-based alternatives, plant-based milk alternatives, supplements and different kinds of energy drinks are not captured in the 2019 Food Composition Table, so package labels were used to assess B_12_ concentrations.

### Sample collection and biochemical analyses

A non-fasting blood sample was collected from all participants. Blood for serum analyses (B_12_, folate and MMA) was collected in a 5·0 ml tube (BD vacutainer SST II advance, Becton Dickinson), and blood for the plasma analysis (tHcy) was collected using a 5·0 ml tube (BD vacutainer PPT K2E 9·0 mg, Becton Dickinson).

The serum tubes were mixed gently by five inversions and placed in a rack at room temperature for 30 to 120 min before centrifugation at 1500 rpm for 10 min (Centrifuge 5804, Eppendorf). All samples were protected from light. The plasma tubes were mixed gently and placed in a rack at room temperature to prevent blood cells attaching to the stopper. The plasma was obtained by the same centrifugation step as above and separation within 30 min of the blood draw. All serum and plasma samples were refrigerated (4°C) until analysis within 3 d at the Fürst Medical Laboratory. The assays were performed using the ADVIA Centaur XP (JEOL Ltd) and XPT System (Siemens Healthiness) by immunoassays coupled with chemiluminescence detection according to the manufacturer’s protocol.

### Deficiency cut-offs and cB_12_ status indicator

B_12_ status was evaluated using the following cut points for serum B_12_ concentrations: severely deficient (≤ 148 pmol/l), marginally deficient (149 to 221 pmol/l), deficient (< 221 pmol/l) and adequate (> 221 pmol/l)^(21)^. Folate deficiency was defined as serum concentrations < 10 nmol/l, adequacy at 10–45 nmol/l and elevated at 45 nmol/l. Elevated tHcy was defined as > 15 µmol/l^(22)^ and elevated MMA as ≥ 0·27 µmol/l^(9)^. The recommended daily intake (RDI) of B_12_ vary across countries. The Nordic Nutrition Recommendations of B_12_ is 2·0 µg/d,^(23)^ and the RDA for the USA is 2·4 µg/d.

The cB_12_ indicator was calculated for participants for which B_12_, tHcy and MMA concentrations were available^(19)^. This approach calculates cB_12_ as a combined indicator of B_12_ status, which can be estimated using two, three or four B_12_ biomarkers (B_12_, tHcy, MMA and holotranscobalamin). cB_12_ values are classified as follows: probable B_12_ deficieny (cB_12_ < –2·5), possible B_12_ deficiency (–2·5 to –1·5), low B_12_ (–1·5 to –0·5), B_12_ adequacy (–0·5 to 1·5) and elevated B_12_ status (cB_12_ > 1·5)^(19)^.

### Ethical approval

This study was conducted according to the guidelines laid down in the Declaration of Helsinki, and all procedures involving human subjects were approved by the Regional Committee for Medical and Health Research Ethics, 2019/653/REC South-East, and the Norwegian Center for Research Data/NSD/101332. Written informed consent was obtained from all participants.

### Statistical analysis

IBM SPSS version 25 (IBM Corp.) was used for the statistical analysis. Normality of the data was tested using visual interpretation of the Q-Q plots and histograms. Spearman’s correlation (r_s_) was used to evaluate the association between continuous non-parametric variables. Correlations below 0·3 were considered to be weak, between 0·3 to 0·5 as moderate and above 0·5 as strong^(24)^. The Mann–Whitney *U* test was used to test differences between groups using non-parametric variables, and the *χ* ^2^ test was used for categorical variables. Cross-tabulations were performed with B_12_, tHcy and MMA as categorical variables to identify potential deficiency. Serum B_12_ was skewed, so all analyses were done using log-transformed data. Multiple linear regression analyses were used to explore predictors of serum B_12_ as the outcome variable. The exposure variables were age, sex, BMI, smoking status, parity, vegan/vegetarian diet, duration of vegan/vegetarian diet, B_12_ intake, B_12_ supplements, B_12_ injections and education. All covariates that showed associations (*P* < 0·10) in the crude regression analysis (age, B_12_ supplements 24 h and total habitual intake of B_12_) were included in the preliminary multiple regression models. Excluded variables were reintroduced, and those that were still associated in this model (age and B_12_ supplements 24 h) (*P* < 0·10) were retained in the final model^(25)^. The regression models were checked for homoscedasticity using standard residuals within ± 3 and Cook’s distance < 1 as parameters.

## Results

### Characteristics of the participants

Fifty-six per cent of the participants were vegans and 44 % were vegetarians (Table 1). Overall, 86 % reported adherence to a vegan or vegetarian diet for more than 2 years, while 14 % stated an adherence for more than 10 years. The use of B_12_ supplements was about 1·5-fold higher in vegans than in vegetarians (*P* < 0·03 for all), while no significant difference was observed in the use of B_12_ injections during the last 10 months (*P* = 0·11).

**Table 1.** Background characteristics of participating vegans and vegetarians in Norway (*n* 205)*

	Combined		Vegans		Vegetarians		
	*n*	%	*n*	%	*n*	%	*P* †
Participants	205	100	115	56	90	44	
Sex							0·005*
Females	148	72·2	74	64·3	74	82·2	
Males	57	27·8	41	35·7	16	17·8	
Country of origin							0·91
Norway	170	83·0	96	83·5	74	82·2	
Other	35	17·0	19	16·5	16	17·8	
Age (years)							
Mean	30·4		31		29·6		0·09
sd	9·1		8·7		9·5		
BMI (kg/m^2^)							
Mean	23·2		23		23·4		0·64
sd	3·5		2·9		3·9		
Educational level							0·65
< 12 years	6	2·9	3	2·6	3	3·3	
12 years	36	17·6	22	19·1	14	15·6	
1–4 years university	165	79·5	90	78·3	73	81·1	
Smoking status							0·93
No	185	90·2	103	89·5	82	91·1	
Yes	20	9·8	12	10·5	8	8·9	
Duration of plant-based diet							
Mean	4·7		4·11		5·5		0·007
sd	3·1		2·8		3·5		
Supplement use							
B_12_, 24-h recall	120	58·5	70	60·9	50	55·6	0·45
B_12_, habitual use	158	77·1	103	89·6	55	61·1	< 0·001*
Folate, habitual use	93	45·4	60	52·2	33	36·7	0·027

* Results are presented in mean ± sd or *n* (%).

† Significant differences as determined by Mann–Whitney *U* test.

### B_12_ intake and status

Median total habitual B_12_ intake (food plus supplements) was higher in vegans compared with vegetarians (*P* < 0·001), while habitual B_12_ intake from foods only was higher in vegetarians (*P* = 0·001, Table 2), so no differences were found in 24-h dietary intake of B_12_. More vegetarians than vegans (40·0 *v*. 18·3 %, *P* < 0·001) had a total habitual B_12_ intake below the RDI of 2 µg/d. No differences in dietary practice affecting in B_12_ intake were found for the 24-h dietary recall. There were no significant differences in B_12_, tHcy or MMA concentrations (medians: B_12_, 357 pmol/l; tHcy, 9·0 µmol/l; MMA, 0·18 µmol/l) between vegans and vegetarians (Table 3).

**Table 2.** Calculated 24-h intake of B_12_, and habitual intake and injection of B_12_ in vegans (*n* 115) and vegetarians (*n* 90)* in Norway

Intakes	Vegans		Vegetarians		
	Median	IQR	Median	IQR	*P* †
B_12_					
24-h intake from food (µg/d)	0·04	0·00, 0·53	0·19	0·01, 0·50	0·57
Total 24-h intake (µg/d)	9·0	1·3, 25·1	6·3	0·50, 25·0	0·64
24-h intake below RDI					
*n*	34		35		0·16
%	29·6		38·9		
Habitual intake from food (µg/d)	0·39	0·17, 0·57	0·52	0·25, 0·74	0·005
Total habitual intake (µg/d)	10·6	2·4, 100	2·7	0·90, 10·4	0·001
Habitual intake < RDI					
*n*	21		36		< 0·001
%	18·3		40·0		
Injections					
*n*	14		5		0·10
%	12·1		5·6		
Time since last injection, months	9·8	4·4, 1–12	10·6	3·1, 5–12	0·80

B_12_, vitamin B_12_; RDI, recommended daily intake.

* Results presented as median (IQR). Total B_12_ intake includes food, energy drinks and supplement use. RDI for B_12_ = 2·0 µg/d^(38)^.

† *P*-values determined by Mann–Whitney *U* test and *χ* ^2^ test for categorical variables.

**Table 3.** Concentrations and deficiency rates of measured blood biomarkers in Norwegian vegans (*n* 115) and vegetarians (*n* 90)*

	Combined		Vegans		Vegetarians			
Markers	*n*	%	*n*	%	*n*	%	*P* †	Ref. values‡
B_12_ (pmol/l)§								
Median	357		350		359		0·88	150–650
IQR	270, 464		288, 450		255, 489			
Deficient	14·3	29	12·4	14	16·7	15	0·43	< 221
Marginal	13·3	27	11·5	13	15·6	14	0·43	149–221
Severe	1·0	2	1·0	1	1·1	1	0·43	< 148
tHcy (µmol/l)||								
Median	9·0		9·0		8·4		0·25	5·0–15·0
IQR	7·1, 11·2		7·7, 11·0		6·4, 11·5			
Elevated	7·0	14	5·3	6	9·1	8	0·44	> 15·0
MMA (µmol/l)¶								
Median	0·18		0·2		0·2		0·55	< 0·27
IQR	0·15, 0·24		0·2, 0·3		0·1, 0·2			
Elevated	19·0	37	19·3	21	16	18·6	0·70	> 0·27
Folate (nmol/l)**								
Median	24·2		25·8		21·6		0·027	6–20
IQR	18·0, 32·9		20·3, 34·8		16·2, 31·3			
Deficient	1·7	3	1·0	1	2·4	2	0·46	< 10·0
cB_12_ ††								
Median	1·30		1·31		1·29		0·11	0·5–1·5
IQR	1·24, 1·36		1·26, 1·36		1·23, 1·36			
Elevated B_12_	3·7	7	2·1	4	1·6	3	0·66	> 1·5

Ref., reference; B_12_, vitamin B_12_; tHcy, homocysteine; MMA, methylmalonic acid; cB_12_, combined B_12_ indicator.

* Values are presented as median (IQR) or % (*n*).

† Mann–Whitney *U* test and *χ* ^2^ test for categorical variables.

‡ Reference values^(19,21)^.

§ B_12_, (vegans, *n* 113; vegetarians, *n* 90).

|| Hcy (vegans, *n* 113; vegetarians, *n* 88).

¶ MMA (vegans, *n* 109; vegetarians, *n* 86).

** Folate (vegans, *n* 98, vegetarians, *n* 82).

†† cB_12_ (vegans, *n* 105; vegetarians, *n* 84). Only participants with B_12_, tHcy and MMA data are included. cB_12_ is dimensionless. None of the cB_12_ values indicated a low status (cB_12_ < −0·5).

The prevalence of B_12_ deficiency (< 221 pmol/l) was 14·3 % (no differences between vegans and vegetarians, *P* = 0·424) based on serum B_12_ concentrations (60 % of these did not have elevated tHcy or MMA). One vegan and one vegetarian had severe B_12_ deficiency (B_12_ ≤ 148 pmol/l). Vegetarian B_12_ supplement users had a higher mean serum B_12_ concentration compared with vegetarian non-users (*P* = 0·002), which was not the case for vegans (online Supplemental Table 1), while supplement use only increased intake in the last 24 h (*P* < 0·001 for all), not habitual B_12_ intake in either group. The overall cB_12_ median value (1·3) was in the adequate B_12_ status range (–0·5 to 1·5), and the cB_12_ values did not differ between vegans and vegetarians (*P* = 0·66). While none of the calculated cB_12_ values fell into the low B_12_ status category for cB_12_ (Table 3), 3·7 % of all participants revealed a cB_12_ value indicating elevated B_12_ (vegans: 2·1 % and vegetarians: 1·6 %).

### Folate supplement use and status

Following the trend of higher supplement use among the vegans (Table 2), vegans had higher serum folate status than vegetarians (25·8 *v*. 21·6 nmol/l, *P* = 0·027, Table 3), and only one vegan and two vegetarians revealed folate levels below the deficiency cut-off of 10 nmol/l (Table 3). Folic acid supplement use increased serum folate in vegans and vegetarians compared with the non-supplement users in each group (*P* ≤ 0·031 for all, Supplemental Table 1).

### Associations among biomarkers

**Figure 1.**
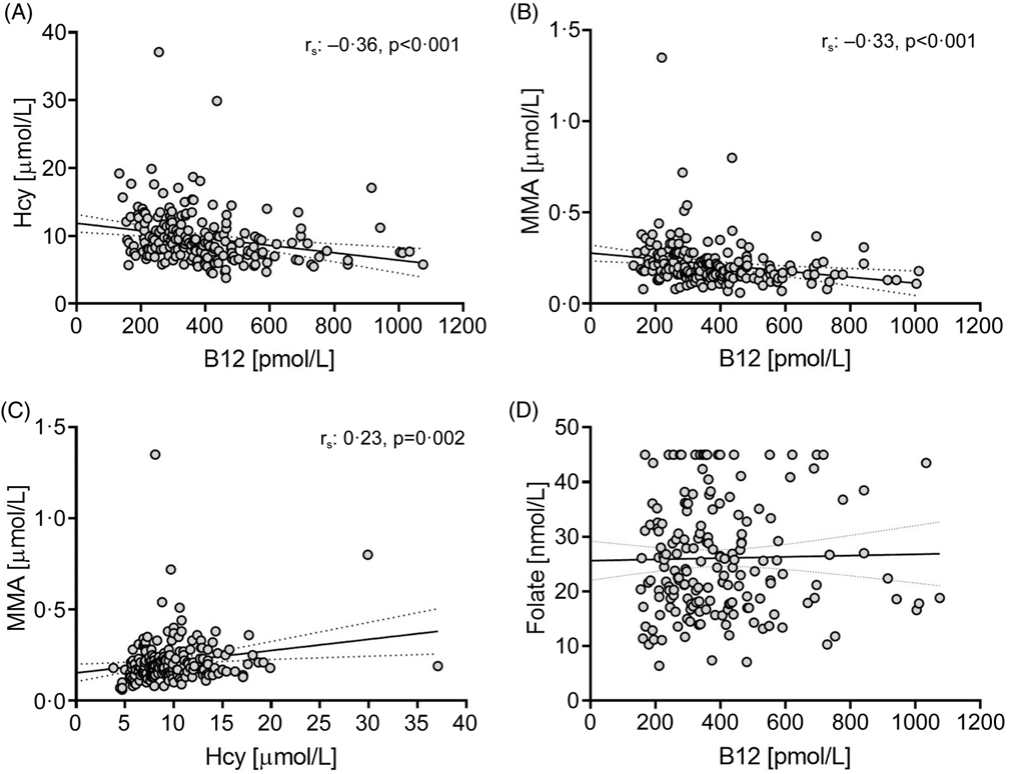
Scatterplots of B12 and related biomarkers in vegans and vegetarians in Norway (n = 205).

Since no significant differences were observed for B_12_, tHcy and MMA concentrations based on dietary practice, the pooled sample set (vegans and vegetarians) was used to examine their relationships. B_12_ concentrations were moderately negatively associated with tHcy and MMA (tHcy, r_s_: −0·36; MMA, r_s_: −0·33, *P* < 0·001 for all), while tHcy and MMA were weakly correlated (r_s_: 0·23, *P* = 0·002; Fig. 1). None of these biomarkers were correlated with folate serum concentrations, regardless of using the pooled or diet-based sample sets. Association between B_12_ biomarkers and folate serum concentration have, however, been found in omnivore populations^(26,27)^.

Supplemental Table 2 shows the percentage of participants with B_12_, tHcy and MMA concentrations (*n* 189) below the respective cut-offs. In total, 75·9 % (*n* 123) of participants with adequate B_12_ status were within the normal range for tHcy and MMA concentrations. The trend was the same for B_12_ deficient and adequate groups: more participants had elevated MMA with normal tHcy (deficient, 29·6 %; adequate, 16·7 %) than normal MMA with elevated tHcy (deficient, 3·7 %; adequate, 6·8 %). Only two participants had low serum B_12_ in conjunction with elevated tHcy and MMA, which was also found in one participant with adequate B_12_ concentrations.

### Predictors of serum B_12_ concentration

In multiple linear regression analysis, increasing age (0·19 (0·001, 0·059), *P* = 0·002) and intake of B_12_ supplement the last 24 h (yes/no) (0·21 (0·013, 0·063), *P* = 0·001) were predictors of serum B_12_ concentration in vegans and vegetarians (Table 4).

**Table 4.** Predictors of B_12_ concentrations in vegans and vegetarians (*n* 205) in Norway

B_12_ predictor	Unadjusted coefficient*	95 % CI	*P*	Adjusted coefficient†	95 % CI	*P*-value
Age	0·004	0·001, 0·006	0·008	0·19	0·001, 0·059	0·002
Supplement use‡	0·078	0·028, 0·13	0·003	0·21	0·013, 0·063	0·001

* The exposure variables were age, sex, BMI, smoking status, parity, dietary practice, duration of dietary practice, B_12_ intake, B_12_ supplements, B_12_ injections and education.

† Adjusted for age, B_12_ supplements 24 h and total habitual intake of B_12_.

‡ Supplement use based on 24-h recall (yes/no).

## Discussion

To the best of our knowledge, this is the first study to assess B_12_ and folate status in vegetarians and vegans in Norway, employing multiple approaches for B_12_ assessment. Despite the fewer dietary B_12_ sources in strict plant-based diets, the combined indicator cB_12_ suggested that none of the participants was B_12_-depleted and 4 % had elevated B_12_. However, 14 % were B_12_-deficient based on serum B_12_ concentration (< 221 pmol/l) including two individuals in the severely deficient range (≤ 148 pmol/l). These two individuals reported use of B_12_ supplements, which suggest other causes of B_12_ deficiency than inadequate dietary B_12_ intake. Similar deficiency rates were found in the National Health and Nutrition Survey (NHANES) for the US adult population (19–59 years, 9·9–10·0 % *v*. 8·7–10·0 % in our study, using their serum B_12_ cut-off of < 200 pmol/l)^(8)^; however, the reported elevated MMA and tHcy prevalence between 3·9–5·2 % and 2·6–6·6 %, respectively, was about 3- to 4-fold lower compared with our study. Interestingly, our findings for B_12_, tHcy and MMA concentrations were comparable to those of the US elderly population in the same report (≥ 60 years). With age, protein-bound B_12_ in the diet is less efficiently absorbed due to a higher occurrence of atrophic gastritis and intestinal bacterial overgrowth^(10)^. In our study, the mean age was 30 years old (with good ability to absorb B_12_), and only three participants were above 60 years old, which may explain the findings of low B_12_ deficiency. While we did not find differences in dietary practice and B_12_ status, other studies have reported lower serum B_12_ concentrations in vegans compared with vegetarians^(28–31)^. However, we found significantly higher total habitual B_12_ intake in vegans (supplement and food), most likely driven by their high doses of B_12_ supplements, which could explain the discrepancy in our findings.

Folate concentrations in nearly all participants were adequate. Good dietary folate sources are fruit and vegetables, and according to Norwegian national dietary surveys, Norwegians have a fruit and vegetable intake below the RDI of 500 g/d^(15,23)^. Correspondingly, the general population in Norway have a lower folate intake than recommended (300 μg/d for adults and 400 μg/d for women of fertile age)^(15,23)^. Since plant-based diets have higher intakes of fruits and vegetables, individuals following these dietary practices might have higher folate status than the general population, a hypothesis supported by our findings of median folate concentrations of over 20 nmol/l compared with the lower values (∼7 to 16 nmol/l) reported in other studies of Norwegian adults (6·7–15·2 nmol/l)^(32)^.

In our study, almost 14 % of the participants were classified as B_12_-deficient based on serum B_12_ concentration. The sensitivity of detecting B_12_ deficiency in its early stages with this biomarker is questionable^(19)^. In fact, cross-tabulation evaluating tHcy and MMA in the B_12_-deficient participants showed that almost 60 % of the B_12_-deficient participants did not have elevated tHcy or MMA. A similar finding was reported in healthy, highly educated vegetarian Indians^(33)^. Furthermore, cB_12_ calculations indicated that none of the participants were low in B_12_. Our study suggests that the deficiency rates of B_12_ are dependent on the method of assessment of B_12_ status, and whether the individuals with low B_12_ concentrations are in fact at risk of deficiency, given that their cB_12_ value indicates adequacy, is doubtful. However, 4 % fell into the elevated cB_12_ range, and 9 % (19) of the participants had a high B_12_ concentration (> 650 pmol/l). A single biomarker like serum B_12_ concentration is not a definitive indicator of B_12_ status or deficiency, if low, it suggests other markers should be used as well. MMA is the most sensitive followed by holoTC, then B_12_ and then homocysteine. cB_12_ includes several of these and is therefore specific and sensitive to detect true deficiency. High doses of B_12_ might be useful to increase serum B_12_ in some situations but given the poor efficiency of absorption of high doses (< 1 %), then either taking a supplement that supplies the usual daily requirement (50 % absorption) or giving a least one dose of i.m. B_12_ might be a better strategy. Surprisingly, supplement use was not the driving factor for such high B_12_ concentrations.

Our findings of inadequate dietary intake of B_12_ in vegans and vegetarians are in agreement with previous reports^(30,34,35)^. Lower habitual dietary B_12_ intake in vegans *v*. vegetarians was also reported in studies from Switzerland and the UK^(29,36)^. Contrarily, adequate B_12_ intake from food in vegans and vegetarians was found in the USA, with nutritional yeast and fortified products as crucial B_12_ sources^(28)^. However, the study also refers to several vegans with insufficient intake. In our study, the main B_12_ sources for vegetarians were fish (consumed by 13 % of the pescatarians) and nutritional yeast (11 % of the total B_12_ intake), emphasising the low consumption of B_12_ from other foods. In Norway, plant-based alternatives for milk, yogurt and cheese are fortified with B_12_; however, no food items are fortified with folic acid.

The use of B_12_ supplements was higher in vegans than vegetarians (71 % *v*. 41 %), which was also reflected in the higher habitual total B_12_ intake in the vegan group. Regardless of the diet, the B_12_ intake was adequate (above RDI) when supplements were taken. While more vegans reported supplement use, the median total B_12_ intake of supplement users did not differ between groups (11·4 *v*. 20·4 µg/d, vegans *v*. vegetarians), reflecting 5- to 10-fold higher intakes than the RDI of 2 µg/d. There is no recommended upper limit for daily B_12_ intake, but there is a proposed maximum intake of 2000 μg as a safety margin^(37)^. Six participants in our study took supplements of B_12_ ≥ 2000 μg, but whether these concentrations adversely affected the participants remains unknown^(38)^. As found in our study, vegans in the USA had highest median intake of B_12_ when supplements were included (9·4 *v*. 6·6 μg/d)^(28)^. Nevertheless, 15 % of vegans and 11 % of vegetarians still had a total B_12_ intake below the RDI, a lower percentage with inadequate intake compared with our study (18 % in vegans and 40 % in vegetarians). In Denmark, the median B_12_ intake of vegans increased from 0 to 17·5 μg/d when B_12_ supplements were included in the diet^(34)^, and a trend also found for the vegans and vegetarians in our study when the 24 h intakes were considered. During the last years, more education about the importance of B_12_ supplements had been made available, especially for vegans, which could explain that most participants reported supplement use.

Only five (5·6 %) vegetarians and fourteen vegans (12·2 %) reported the use of B_12_ injections, which had no effect on the measured serum B_12_ concentrations. Since only four participants reported a B_12_ injection within the last 1 to 5 months, and all remaining injection users received their last injection 12 months prior, the treatment is not likely to significantly alter the results in this study. Moreover, it has been estimated that only about 15 % of a 1000 µg intramuscular B_12_ dose is retained in the body^(9)^, further indicating that the B_12_ injections as reported in this study are no major contributor to B_12_ concentrations. In fact, use of daily high-dose B_12_ supplements (1000–2000 µg) have been reported to be equal or even superior to injections, supporting our findings of the positive effect of supplements on B_12_ concentrations.

We found B_12_ supplement use to be the strongest predictor of serum B_12_ concentrations, and the most important B_12_ source consumed among both vegans and vegetarians in our study. Further, higher B_12_ concentrations were associated with increasing age, which in turn supported a better B_12_ status. The low B_12_ concentrations in a surprisingly high percentage of younger vegan and vegetarian adults may indicate lower compliance with B_12_ supplementation and a higher risk of B_12_ deficiency in this age group. To secure optimal growth and development of the foetus, adequate B_12_ status during pregnancy and lactation is crucial. Of notice, in the Nordic Nutrition Recommendations 2012, the RDI during lactation is raised from 2·0 µg /d to 2·6 µg /d, underlining there is an urgent need to conduct more trials to investigate whether intervention with prenatal and postnatal vitamin B_12_ supplementation would improve child health outcomes in populations at risk.

### Strengths and limitations

The B_12_ dietary intake could be underestimated as the Norwegian Food Composition Table is not fully updated regarding B_12_ content in plant-based food alternatives, such as cheese/milk and yogurt substitutes, or other available B_12_-enriched products. Nonetheless, we registered B_12_ intake manually. A limitation in our study was that dietary folate intake was not calculated. The low number of participants in the group of non-supplement users, among vegans, was also a limitation. A higher sample size could have produced more valuable findings in this regard. The higher rate of educational level of our study participants compared with the general Norwegian population (77 % *v*. 34 %)^(31)^ may have caused our results to be unrepresentative of the Norwegian population, as higher education has been associated with better health and healthier eating habits^(32)^. Moreover, since convenience sampling was used to recruit participants, more vegans and vegetarians may have been included who are extremely concerned about their diet and health.

However, this study analysed multiple biomarkers for B_12_ to evaluate subclinical or clinical B_12_ deﬁciency, namely tHcy, MMA, and erythrocyte folate, and we also calculated and evaluated the combined B_12_ status indicator, cB_12_. The availability of dietary data from 24-h recall and FFQ allows us to map B_12_ dietary sources and supplements. In addition, we had low percentage of missing data due to the use of electronic questionnaires with mandatory answer options.

### Conclusions

This is the first study in vegans and vegetarians in Norway to assess B_12_ and folate status, using multiple approaches for its assessment. Despite fewer dietary B_12_ sources in strict plant-based diets, most participants revealed adequate B_12_ status due to B_12_ supplementation. Both vegans and vegetarians had adequate folate status, indicating adequate folate intake.

## Supplementary material

For supplementary material accompanying this paper visit https://doi.org/10.1017/S0007114522002987. click here to view supplementary material
