# Supplementary material for: Adequate vitamin B12 and folate status of Norwegian vegans and vegetarians
Source: Br J Nutr. 2022 Sep 27;129(12):2076–83. doi: 10.1017/S0007114522002987 (PMC10197078; doi:10.1017/S0007114522002987)
Supplement: Supplementary file 1 [file S0007114522002987sup001.docx]

**Supplemental Table 1:** B12 and folate median concentrations (IQR) and total intakes of vegan and vegetarian supplement and non-supplement users in Norway

|  | **No supplement** | **Supplement** | **p-value^1^** |
| --- | --- | --- | --- |
| Combined, n (%) | 84 (41.2) | 120 (58.8) |  |
| B12, pmol/L | 313 (249, 406) | 399 (296, 503) | 0.002 |
| B12 24h intake, µg/day | 0.48 (0.01, 1.6) | 18.9 (10.0, 50.5) | <0.001 |
| B12 habitual intake, µg/day | 5.6 (1.7, 25.1) | 5.5 (1.4, 51.4) | 0.48 |
| Folate, nmol/L | 21.7 (17.0, 28.0) | 29.0 (20.2, 40.5) | <0.001 |
| Vegetarian, n (%) | 40 (44.4) | 50 (55.6) |  |
| B12, pmol/L | 292 (243, 376) | 427 (296, 576) | 0.002 |
| B12 24h-intake, µg/day | 0.42 (0.10, 1.5) | 20.4 (10.0, 50.0) | <0.001 |
| B12 habitual intake, µg/day | 4.2 (0.95, 12.8) | 2.4 (0.90, 9.8) | 1.00 |
| Folate, nmol/L | 20.8 (15.8, 27.3) | 29.0 (17.7, 40.5) | 0.031 |
| Vegan, n (%) | 44 (38.6) | 70 (61.4) |  |
| B12, pmol/L | 322 (279, 420) | 379 (296, 473) | 0.17 |
| B12 24h-intake, µg/day | 0.48 (0.0, 2.0) | 11.4 (10.0, 51.0) | <0.001 |
| B12 habitual intake, µg/day | 9.8 (3.8, 26.4) | 12.8 (2.4, 100) | 0.46 |
| Folate, nmol/L | 22.5 (18.0, 29.9) | 28.8 (21.7, 42.4) | 0.002 |

^1^ Generalized linear model. IQR, interquartile range.

**Supplemental Table 2:** Percentage of participants with B12, tHcy and MMA concentrations (n=189) below cut-off of vegans and vegetarians in Norway (n = 189)^1^

| **B12 status indicators** | **B12** | |
| --- | --- | --- |
|  | Deficient  n (%) = 27 (14.3) | Normal  n (%) = 162 (85.7) |
| tHcy normal, MMA normal , *n (%)* | 16 (59.3) | 123 (75.9) |
| tHcy normal, MMA elevated, *n (%)* | 8 (29.6) | 27 (16.7) |
| tHcy elevated, MMA normal, *n (%)* | 1 (3.7) | 11 (6.8) |
| tHcy elevated, MMA elevated,  *n (%)* | 2 (7.4) | 1 (0.6) |
| Folate, deficient, *n (%)* | 0 (0.0) | 1 (0.6) |
| p-value^2^ | 0.015 | |

^1^B12 deficiency defined as B12 <221 pmol/L, normal B12 as ≥221 pmol/L. Elevated tHcy defined as >15 µmol/L, elevated MMA as ≥27 µmol/L, folate deficiency as <10nmol/L.

^2^chi-squre test. B12, vitamin B12; tHcy, homocysteine; MMA, methyl malonic acid.

**Supplemental table 3**: Food groups in the food frequency questionnaire

| **Dietary food groups** | **Frequency alternatives** |
| --- | --- |
| Bread/ crisp rolls | Seven frequency options, ranging from rare/never to ≥5 times a day |
| Cereals, unsweetened | Seven frequency options, ranging from rare/never to ≥5 times a day |
| Cereals, sweetened | Seven frequency options, ranging from rare/never to ≥5 times a day |
| Rice/pasta | Seven frequency options, ranging from rare/never to ≥5 times a day |
| Cow`s milk | Seven frequency options, ranging from rare/never to ≥5 times a day |
| Plant-based milk alternatives, all types | Seven frequency options, ranging from rare/never to ≥5 times a day |
| Yoghurt, based on cow`s milk | Seven frequency options, ranging from rare/never to ≥5 times a day |
| Plant-based yoghurt alternatives | Seven frequency options, ranging from rare/never to ≥5 times a day |
| Fatty fish | Seven frequency options, ranging from rare/never to ≥5 times a day |
| Lean fish | Seven frequency options, ranging from rare/never to ≥5 times a day |
| Fish products | Seven frequency options, ranging from rare/never to ≥5 times a day |
| Sushi | Seven frequency options, ranging from rare/never to ≥5 times a day |
| Meat replacement | Seven frequency options, ranging from rare/never to ≥5 times a day |
| Legumes | Seven frequency options, ranging from rare/never to ≥5 times a day |
| Olive oil (salad/cooking) | Seven frequency options, ranging from rare/never to ≥5 times a day |
| White cheese | Seven frequency options, ranging from rare/never to ≥5 times a day |
| Vegan cheese | Seven frequency options, ranging from rare/never to ≥5 times a day |
| Whey cheese | Seven frequency options, ranging from rare/never to ≥5 times a day |
| Eggs | Seven frequency options, ranging from rare/never to ≥5 times a day |
| Cakes, chocolate, ice cream (dairy based) | Seven frequency options, ranging from rare/never to ≥5 times a day |
| Salty snacks | Seven frequency options, ranging from rare/never to ≥5 times a day |
| Sweetened drinks | Seven frequency options, ranging from rare/never to ≥5 times a day |
| Artificially sweetened drinks | Seven frequency options, ranging from rare/never to ≥5 times a day |
| Water (as beverage) | Seven frequency options, ranging from rare/never to ≥5 times a day |
| Coffee | Seven frequency options, ranging from rare/never to ≥5 times a day |
| Tea | Seven frequency options, ranging from rare/never to ≥5 times a day |
| Energy drink | Seven frequency options, ranging from rare/never to ≥5 times a day |
| Sparkling water | Seven frequency options, ranging from rare/never to ≥5 times a day |
| Vegetables, all types | Seven frequency options, ranging from rare/never to ≥5 times a day |
| Fruits, all types | Seven frequency options, ranging from rare/never to ≥5 times a day |
| Potatoes | Seven frequency options, ranging from rare/never to ≥5 times a day |
| Nuts (all types, not salted) | Seven frequency options, ranging from rare/never to ≥5 times a day |
